# Role of a Novel PH-Kinase Domain Interface in PKB/Akt Regulation: Structural Mechanism for Allosteric Inhibition

**DOI:** 10.1371/journal.pbio.1000017

**Published:** 2009-01-20

**Authors:** Véronique Calleja, Michel Laguerre, Peter J Parker, Banafshé Larijani

**Affiliations:** 1 Cell Biophysics Laboratory, Cancer Research UK, Lincoln's Inn Fields Laboratories, London Research Institute, London, United Kingdom; 2 UMR 5248-CNRS, Institut Européen de Chimie et Biologie, Pessac, France; 3 Protein Phosphorylation Laboratory, Cancer Research UK, Lincoln's Inn Fields Laboratories, London Research Institute, London, United Kingdom; 4 Division of Cancer Studies KCL, Guy's Hospital, London, United Kingdom; Beth Israel Deaconess Medical Center, United States of America

## Abstract

Protein kinase B (PKB/Akt) belongs to the AGC superfamily of related serine/threonine protein kinases. It is a key regulator downstream of various growth factors and hormones and is involved in malignant transformation and chemo-resistance. Full-length PKB protein has not been crystallised, thus studying the molecular mechanisms that are involved in its regulation in relation to its structure have not been simple. Recently, the dynamics between the inactive and active conformer at the molecular level have been described. The maintenance of PKB's inactive state via the interaction of the PH and kinase domains prevents its activation loop to be phosphorylated by its upstream activator, phosphoinositide-dependent protein kinase-1 (PDK1). By using a multidisciplinary approach including molecular modelling, classical biochemical assays, and Förster resonance energy transfer (FRET)/two-photon fluorescence lifetime imaging microscopy (FLIM), a detailed model depicting the interaction between the different domains of PKB in its inactive conformation was demonstrated. These findings in turn clarified the molecular mechanism of PKB inhibition by AKT inhibitor VIII (a specific allosteric inhibitor) and illustrated at the molecular level its selectivity towards different PKB isoforms. Furthermore, these findings allude to the possible function of the C-terminus in sustaining the inactive conformer of PKB. This study presents essential insights into the quaternary structure of PKB in its inactive conformation. An understanding of PKB structure in relation to its function is critical for elucidating its mode of activation and discovering how to modulate its activity. The molecular mechanism of inhibition of PKB activation by the specific drug AKT inhibitor VIII has critical implications for determining the mechanism of inhibition of other allosteric inhibitors and for opening up opportunities for the design of new generations of modulator drugs.

## Author Summary

A critical protein in cell-signalling pathways, called protein kinase B, regulates many aspects of cell biology from metabolism to proliferation and survival, by modifying other proteins with the addition of a phosphate group. Hence, deregulation of its activity has acute consequences on cell function. Increased activity of a tumour-promoting form of protein kinase B or of upstream regulatory proteins has been observed in tumours, while impaired protein kinase B function has been linked to diabetes. Therefore, understanding the molecular mechanism of protein kinase B activation will help reveal how its activity might be regulated to limit disease progression. Toward this end, we studied how protein kinase B structure relates to its function, to identify molecular mechanisms regulating its kinase activity, modifying its cellular localization, and altering its binding to other proteins. By determining the spatial organization of different regions of the protein in inactive protein kinase B, we discovered a cavity at the interface of two distinct functional regions of the inactive form. We also localized the C-terminal end of the protein to the apex of the cavity, suggesting a role of this domain in regulating the inactive form of the protein. This represents a novel example of negative regulation by inhibition across these different regions of the protein. From these findings, we elucidated the mechanism of action of a highly specific protein kinase B inhibitor, AKT inhibitor VIII. We determined that simultaneous binding of the inhibitor to the two different functional regions, through the cavity, “locks” protein kinase B in an inactive conformation and prevents regulatory proteins from accessing the C-terminal domain.

## Abstract 3

The mechanism of inhibition of the activity of the tumor-promoter protein kinase B operates via interactions across its functional domains.

## Introduction

Protein kinase B (PKB/Akt) is a key regulator downstream of various growth factors and hormonal signals. It activates a panel of proteins that regulate proliferation, growth, survival, or metabolism and is involved in human cancer [1,2]. In particular, its overexpression induces malignant transformation and chemoresistance [3]. PKB belongs to the AGC superfamily of related serine/threonine protein kinases. Three isoforms of PKB exist in mammals (PKBα/Akt1, PKBβ/Akt2, and PKBγ/Akt3) that comprise an N-terminal pleckstrin homology (PH) domain, a flexible hinge between the PH and the kinase domain, a catalytic (kinase) domain, and a C-terminal regulatory part (containing a hydrophobic motif, or HM) (for review [4,5]). The phosphorylation of Thr 308 in the kinase domain of PKBα/Akt 1 by phosphoinositide-dependent protein kinase-1 (PDK1) [6] and Ser 473 in the hydrophobic motif by mTORC2 complex [7] and/or DNAPK [8], is central for PKB activation [9]. These phosphorylations were shown to be dependent on the colocalisation of PKB and PDK1 at the plasma membrane through the interaction of their PH domains with PtdIns (3,4,5) P_3_ and PtdIns (3,4) P_2_ for the former [10–12] and PtdIns (3,4,5) P_3_ for the latter [13]. The dephosphorylation of these residues by okadaic acid-sensitive and -insensitive phosphatases was shown to deactivate PKB [14,15].

Recently, the cytoplasmic interaction of inactive PKB with PDK1 was published [14]. The maintenance of PKB in an inactive conformation by the interaction of its PH and kinase domains (PH-in) prior to stimulation prevented the phosphorylation of Thr 308 by the associated PDK1. Upon stimulation, PKB PH-domain interaction with phosphoinositides and its concomitant change in conformation allowed the separation of the PH and kinase domains and the associated co-recruited PDK1 to phosphorylate Thr 308.

Here, the intramolecular interactions of the PKB domains in its inactive conformation were studied in detail using molecular dynamics in conjunction with classical biochemical approaches and in situ Förster resonance energy transfer (FRET)/two-photon fluorescence lifetime imaging microscopy (FLIM). A PH domain-induced cavity was discovered in the inactive PKBα kinase domain. The formation of this structure was dependent on the PH domain residue Trp 80. The position of PKBα C-terminal HM at the apex of the PH domain-induced cavity in the inactive conformation of PKB was also described. Unlike in PKBα, a PH domain-induced cavity did not appear in PKBγ suggesting a potential role of this structure in a differential regulation of these two PKB isoforms.

These findings led to the elucidation of the molecular mechanism of inhibition of PKB by the highly specific allosteric inhibitor, AKT inhibitor VIII, and its relationship to the C-terminal HM motif of PKB.

## Results and Discussion

**Figure 1:**
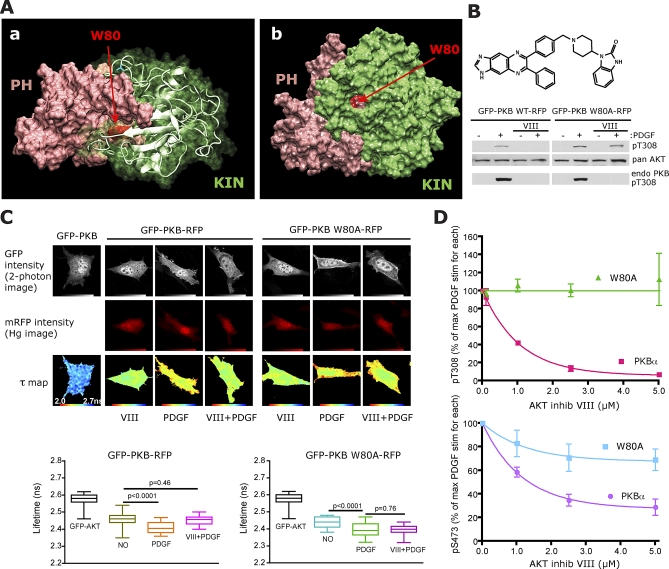
In PKBα Inactive Conformation the PH Domain Creates a PH Domain-Induced Cavity in the Kinase Domain, Binding Site for AKT Inhibitor VIII (A) The structure of the PKBα PH domain (pink) and reconstructed kinase domain (green) is shown in panel (a) in complex (PKBα PH-in conformer) after short dynamic runs and minimisation in water and physiological salt concentration. The secondary structure of the kinase domain is represented as a white ribbon. The PH domain residue Trp 80 located deep inside the kinase domain cleft is shown in red. The image in panel (b) was obtained by rotation of 90° about the *x*-axis of the image panel (a). From this view, Trp 80 (red) is visible through an open cavity in the kinase domain. (B) NIH3T3 cells were transfected with GFP-PKBα-RFP wt or Trp 80 Ala mutant constructs. The next day, the cells were treated with 50 μM of AKT inhibitor VIII for 30 min prior to 5-min PDGF stimulation as indicated. The expression of the fusion proteins and their phosphorylation on Thr 308 were determined by western blotting with an anti-PKB antibody (pan Akt) and a phosphospecific antibody, respectively. Thr 308 phosphorylation of endogenous PKB is shown in the lower panel. The chemical structure of AKT inhibitor VIII is represented above the western blot. (C) Two-photon FLIM images of NIH3T3 cells transfected either with GFP-PKBα or GFP-PKBα-mRFP wt or Trp 80 Ala mutant as indicated. The top panels present intensity images of the donor GFP chromophore in the transfected cells after two-photon excitation. The central panels present intensity images of the acceptor chromophore mRFP obtained with a mercury (Hg) lamp. The bottom panels present the calculated lifetime maps of the donor GFP-PKBα or GFP-PKBα-mRFP. The lifetimes are presented in a pseudo-colour scale going from 2.0 ns (red) to 2.7 ns (blue). The cells were treated with 50 μM of AKT inhibitor VIII for 30 min prior to 5-min PDGF stimulation as indicated. The statistical distributions of lifetimes in each condition are shown as box and whiskers plots: GFP-PKBα-mRFP (left graph) and Trp 80 Ala (right graph). The statistical analysis was performed on at least 15 cells in three independent experiments. The *p-*values between two conditions are indicated on the graphs. (D) The graphs present the effect of a dose response of AKT inhibitor VIII on Thr 308 (upper graph) and Ser 473 (lower graph) phosphorylations of GFP-PKBα wt and Trp 80 Ala mutant. The values plotted have been normalised to 5-min PDGF stimulation for each constructs. The quantifications were performed using the Odyssey/LI-COR fluorescence detection system.

Previously, the molecular model of the inactive PKB conformer (PH-in conformer) showed that the PH and kinase domains interacted [14]. By using molecular dynamics, an unpredicted formation of a PH-induced cavity in the PKB kinase domain was observed (Figure 1A). The location of this cavity appeared to be in the same region as the PKB hydrophobic pocket (binding site for PKB HM) described in the crystal structure of the isolated PKB kinase domain [16]. The initial docking model of the PKB PH and kinase domains interaction prior to performing molecular dynamics revealed that the residue Trp 80, critically positioned at the tip of the VL3 loop of the PH domain, was buried deep inside the PKB kinase domain cleft in the PH-in conformer. However, the refined dynamic model showed that through this PH-induced cavity, the Trp 80 residue, located at its centre, was accessible from outside, whereas PKB remained in its inactive conformer (Figure 1A-b).

To illustrate the importance of the positioning of the Trp 80 and the PH-induced cavity, we took advantage of a specific PKB inhibitor, AKT inhibitor VIII [17–19] (Figure 1B, chemical structure). The selectivity has been attributed to an allosteric mode of binding, noncompetitive with ATP or substrates, and with a mechanism of action dependent on the presence of the PH domain of the full-length protein. In vivo AKT inhibitor VIII prevented the phosphorylation of Thr 308 and Ser 473 (Figure 1B, western blot, and [20]), and as a result, it was proposed to target a specific PKB conformation. Recently, its efficacy was shown to be dependent on the presence of the Trp 80, but the molecular mechanism was not determined [21]. The targeting of the PKB inactive conformer by AKT inhibitor VIII and consequent prevention of PKB conformation change was a plausible explanation for the loss of Thr 308 phosphorylation.

To verify this postulate in vivo, experiments with the PKB fluorescent probe (green fluorescent protein [GFP]-PKB-monomeric red fluorescent protein [mRFP]) [14] were performed. The platelet-derived growth factor (PDGF)-induced change in the conformation of PKB was monitored by FRET/two-photon FLIM (Figure 1C). The pretreatment of NIH3T3 cells with AKT inhibitor VIII prevented both the translocation of GFP-PKB-mRFP to the plasma membrane and its change in conformation induced by PDGF. To illustrate the importance of the interaction of the inhibitor with Trp 80, the GFP-PKB W80A-mRFP mutant was used. Upon PDGF stimulation, the W80A mutant behaved as wild-type PKB. However, in response to PDGF, it was evident that the mutant was not inhibitor sensitive. The graphs in Figure 1C illustrate the statistical data obtained by FRET/two-photon FLIM. These results established that the inhibitor “locked” PKB in its PH-in conformer and confirmed in vivo the importance of the Trp 80 residue in this mechanism.

The inhibition of PDGF-induced Thr 308 and Ser 473 phosphorylation by preincubation with increasing doses of AKT inhibitor VIII was tested on GFP-PKBα and on the Trp 80 mutant (Figure 1D). At 5 μM AKT inhibitor VIII, the phosphorylation of Thr 308 was inhibited by 95%, but Ser 473 was inhibited by only 70%. This differential in the inhibition of Thr 308 phosphorylation compared to Ser 473 indicated that the mechanism of action of the inhibitor affected the two sites in a distinct manner. The mutation of the Trp 80 to Ala prevented entirely the loss of Thr 308 phosphorylation induced by 5 μM inhibitor, confirming that the loss of this critical residue prevented the locking of PKB in the PH-in conformation as observed in Figure 1C. However, the possibility that the inhibitor might still interfere with the second site phosphorylation could not be excluded since a slight decrease of Ser 473 phosphorylation was observed in the W80A mutant.

**Figure 2:**
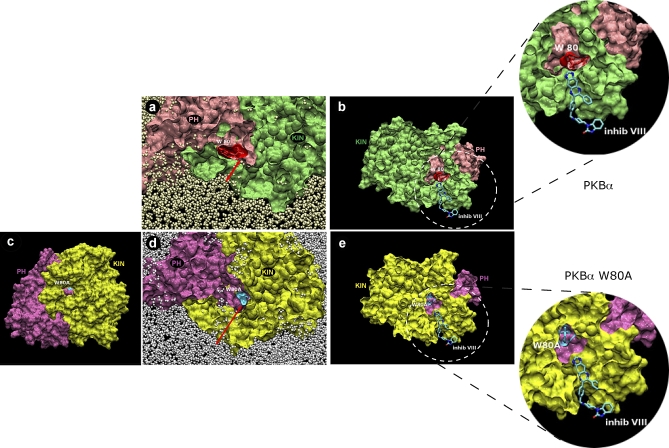
Model of AKT Inhibitor VIII Positioned in the PH Domain-Induced Cavity The image in panel (a) is a vertical cross section of the dynamic structure shown in Figure 1A at the level of the PH-induced cavity. The distance between the Trp 80 residue in red and the protein surface is about 8–8.5 Å (red arrow). The cavity is filled with water molecules shown as khaki pearls. In panel (c), the dynamic structure of the PH domain mutant Trp 80 Ala (purple) interacting with the wild-type kinase domain (yellow) is shown from the same angle as in Figure 1A-b. The Ala 80 in light blue is positioned differently compared to Trp 80. On the vertical cross section (d), the PH-induced cavity is not properly formed. Fewer water molecules accommodate the cavity (grey pearls), and the distance between Ala 80 and the surrounding water environment has increased (14–15 Å) compared to Trp 80 (red arrows). The modelled positions of AKT inhibitor VIII (blue chemical structure) in the PH-induced cavity of PKBα and PKBα Trp 80 Ala mutant are shown in panels (b) and (e), respectively.

To understand the significance of the Trp 80 residue in PKB, the interaction of PKBα PH mutant W80A with the kinase domain in the PH-in conformation was determined by molecular dynamics (Figure 2-c and 2-d). The alteration of the shape and length of the PH domain-induced cavity in the mutant PKBα W80A as compared to wild-type PKBα is illustrated in Figure 2-c and 2-d (compare to Figures 1A-b, and 2-a). Both cavities are filled with water molecules represented in khaki or grey pearls (Figure 2-a and 2-d). More remarkably, the distance between residue 80 and the protein surface (red arrows) was increased in PKBα W80A (14 Å) compared to wild type (8 Å). Furthermore, the maximum width of the cavity was reduced in the W80A (6–7 Å) compared to the wild type (8–9 Å). Figure 2-b presents the model of interaction of AKT inhibitor VIII with the PH-induced cavity of PKBα. Since the model showed a structure of the inhibitor strongly compatible with the length and the shape of the cavity in PKBα, a molecular simulation of the interaction of the inhibitor with the PKBα PH-in structure (Figure S1) was performed. Following a 1-ns dynamic run, AKT inhibitor VIII was deeply and stably embedded in the cavity of wild type PKBα. A distance of 4 Å measured from the quinoxalin head of AKT inhibitor VIII to Trp 80 suggested a possible interaction by hydrogen bonding. Therefore, the docking of AKT inhibitor VIII in the PH-induced cavity and its interaction with Trp 80 would maintain PKB in the inactive conformer. Conversely, the deformation of the cavity in the Trp 80 Ala mutant (Figure 2-e) and the loss of bonding with the 80 Ala residue, located farther away in the structure, would explain the loss of inhibitor effect.

**Figure 3:**
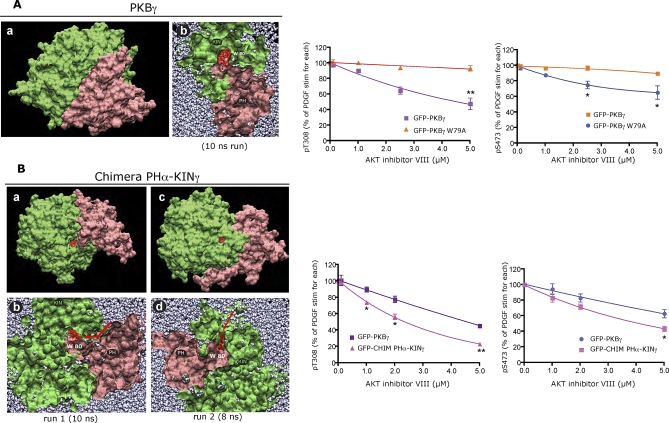
The Lower Sensitivity of PKBγ for AKT Inhibitor VIII Can Be Partially Reversed by Creating a Chimera (PHα-KINγ) (A) A dynamic model of the PH-in conformation of PKBγ after 10-ns run is shown in panels (a) and (b) (cross section at the level of Trp 79). NIH3T3 cells expressing GFP-PKBγ or a mutant in which Trp 79 was changed to an Ala (W79A) were treated for 30 min with different concentrations of AKT inhibitor VIII as indicated prior to 5-min PDGF stimulation. Thr 308 and Ser 473 phosphorylations were detected by western blot using phosphospecific antibodies, and the quantifications were done using the Odyssey/LI-COR fluorescence detection system. The data are presented in percentage of Thr 308 (left graph) or Ser 473 (right graph) phosphorylations upon PDGF stimulation for each construct. (B) Two dynamic runs of the PH-in conformation of the chimera PHα-KINγ of 10 ns and 8 ns are presented in panels (a and b) and (c and d), respectively. The dynamic models show that the replacement of PH and linker of PKBγ by those of PKBα in the chimera PHα-KINγ lead to a partial reopening of the PH-induced cavity in the kinase domain KINγ of the chimera. NIH3T3 cells expressing GFP-PKBγ or GFP-CHIM PHα-KINγ were treated for 30 min at different concentrations of AKT inhibitor VIII as indicated prior to 5-min PDGF stimulation. Thr 308 and Ser 473 phosphorylations were detected by western blot using phosphospecific antibodies and the quantifications were performed using the Odyssey/LI-COR fluorescence detection system. The data are presented as a percentage of Thr 308 (left graph) or Ser 473 (right graph) phosphorylations upon PDGF stimulation for each construct. The *p-*values were calculated using an unpaired *t*-test with Welch's correction on a minimum of three independent experiments. A single asterisk (*) and double asterisks (**) indicate *p* < 0.025 and *p* < 0.001, respectively. Error bars indicate the standard error of the mean.

To understand whether this dynamic model was compatible with the reported selective effect of AKT inhibitor VIII towards PKBα and -β compared to PKBγ [17,20], the reconstruction of a full-length PKBγ based on the PKBα model described above, was made and a dynamic run of PKBγ in the PH-in conformation was performed (Figure 3A). Contrary to PKBα, a PH domain-induced cavity was not formed in PKBγ (Figure 3A-a and 3A-b). However, the dynamic structure revealed that Trp 79 (equivalent to Trp 80 in PKBα) was accessible, since it was positioned close to the surface (6 Å). A dose response of inhibitor VIII indicated a lower sensitivity for PKBγ compared to PKBα. An inhibition of Thr 308 and Ser 473 phosphorylations of only 50% and 30%, respectively, was observed at 5 μM inhibitor (Figure 3A). The mechanism of inhibition also required the presence of the residue Trp 79, as AKT inhibitor VIII failed to inhibit the phosphorylation of Thr 308 and Ser 473 in the mutant GFP-PKBγ W79A. These data indicated that the mechanism of inhibition of PKBγ was specific and similar to PKBα, although less potent. Since the major difference between the two isoforms was the presence of the PH domain-induced cavity, a possible explanation for a reduced sensitivity of PKBγ was that the absence of the cavity impaired the access of the inhibitor to Trp 79.

To further probe this behaviour, a chimera was engineered between the PH domain and linker of PKBα, and the kinase domain and C-terminal part of PKBγ (Figure 3B). Two dynamic runs (8 ns and 10 ns) of the chimera PHα-KINγ in the PH-in conformation were performed (Figure 3B). The partial reopening of an unstable PH domain-induced cavity was observed in the chimera PHα-KINγ (Figure 3B-a through 3B-d). Concomitantly, the sensitivity of the chimera PHα-KINγ for AKT inhibitor VIII was enhanced compared to PKBγ (Figure 3B, right graphs). An inhibition of Thr 308 and Ser 473 phosphorylation of 80% and 55%, respectively, at 5 μM was observed. However, the increased sensitivity of the chimera PHα-KINγ for the inhibitor did not reach the sensitivity of wild-type PKBα. These results suggested a strong correlation between the efficacy of AKT inhibitor VIII and the presence of the PKB PH domain-induced cavity.

In summary, the association of the PH domain residue Trp 80 to AKT inhibitor VIII through the PH domain-induced cavity would block PKB in the inactive PH-in conformer, preventing Thr 308 accessibility and it being phosphorylated. A similar mechanism occurs in PKBγ; however, the reduced accessibility of Trp 79 decreases the efficiency of the inhibitor. To our knowledge, this is the first time the presence of a PH domain-induced cavity in PKBα has been demonstrated.

The loss of PKB conformation change upon inhibitor binding was not a fully satisfactory explanation for the loss of Ser 473 phosphorylation. Therefore, the position of the C-terminal HM of PKB in its PH-in conformation relative to AKT inhibitor VIII binding was investigated using molecular modelling.

**Figure 4:**
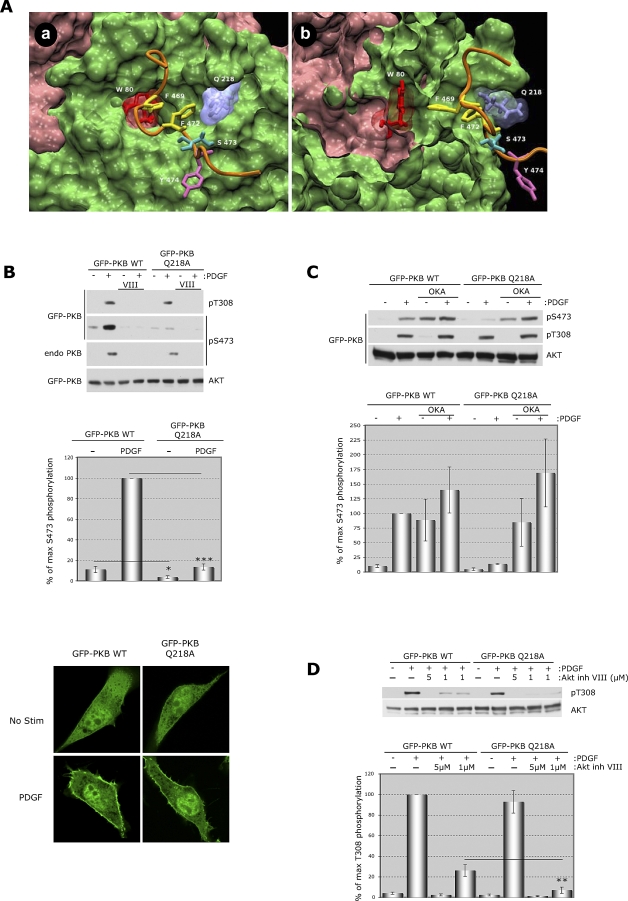
The Position of the C-Terminal Hydrophobic Motif (HM) of PKBα in the PH-in Conformer Is at Close Proximity to its Position after Stimulation and Interferes with AKT Inhibitor VIII Binding (A) PKBα C-terminal hydrophobic motif (HMα) (orange) interacting with PKBα kinase domain (green) in the PH-in conformer is presented in the model panels (a) and (b) (cross section). The PH domain is in pink, and the PH domain residue Trp 80 is in red. The model shows that two phenylalanines (in yellow) from the sequence F_469_XXF_472_S of HM are found positioned right above the PH-induced cavity in PKBα kinase domain. The Phe 469 is at binding distance (∼3.5 Å) from Trp 80 (panel [b]). The principal amino acid residues are indicated. (B) NIH3T3 cells expressing GFP-PKBα wt or a mutant Q218A, were treated for 30 min with 5 μM AKT inhibitor VIII prior to 5-min PDGF stimulation as indicated. Thr 308 and Ser 473 phosphorylations of exogenous and endogenous PKB were detected by western blot using phosphospecific antibodies. Total protein was detected using a pan AKT antibody. The histogram presents the quantification of Ser 473 phosphorylation as a percentage of maximal phosphorylation upon PDGF stimulation in GFP-PKBα wt. A one-sample *t*-test was performed (triple asterisks [***]) showing an extremely significant difference with *p* < 0.0001. An unpaired *t*-test with Welch's correction (single asterisk [*]) shows a very significant difference with a *p* < 0.05. The bottom panels show confocal images at mid-section of NIH3T3 cells expressing GFP-PKBα wt or GFP-PKBα Q218A prior to (No Stim) or upon 5-min PDGF stimulation. endo, endogenous; OKA, okadaic acid. (C) NIH3T3 cells expressing GFP-PKBα wt or the mutant (Q218A), were treated for 45 min with 1 μM of okadaic acid prior to 5-min PDGF stimulation as indicated. In the top panels, Ser 473 and Thr 308 phosphorylations were detected by western blot using phosphospecific antibodies. Total protein was detected using a pan AKT antibody. The histogram presents the quantitative analysis of Ser 473 phosphorylation as a percentage of PDGF stimulation in GFP-PKBα wt, from three independent experiments. (D) NIH3T3 cells expressing GFP-PKBα wt or mutant Q218A were treated for 30 min with different doses of AKT inhibitor VIII (AKT inh VIII) prior to 5-min PDGF stimulation as indicated. Thr 308 phosphorylation was detected by western blot using a phosphospecific antibody. Total protein was detected using a pan AKT antibody. The histogram presents the quantitative analysis of Thr 308 phosphorylation as a percentage of PDGF stimulation in wild type, from eight independent experiments. An unpaired *t*-test with Welch's correction was performed with (double asterisks [**]) representing *p* < 0.005.

The only available crystal structure by Yang et al. (PDB code: 1O6K) [16,22] demonstrated that a modified HM (in which Ser 473 was mutated to an Asp) was positioned in a hydrophobic grove in the N lobe of the isolated active PKBβ kinase domain; the positioning of the HM in inactive full-length PKB has not been investigated. Figure 4A illustrates the positioning the HM of PKBα on the PKBα kinase domain in its PH-in conformer after a simulation of 11 ns, as depicted by the crystal structure [16]. The dynamic model showed that the HM passed right across the PH-induced cavity. Specifically, the sequence F_469_XXF_472_S of HM was kinked above the cavity, thus positioning Phe 469 at a binding distance (3.5 Å) from Trp 80.

From this model, Gln 218 was identified as being at an interacting distance for Ser 473. In addition, this residue appeared to be at about 5 Å from AKT inhibitor VIII in the dynamic interaction model (Figure S1). Therefore, mutagenesis was performed to assess its potential role in the HM position and its relation to the inhibitor binding. By mutating Gln 218 to Ala in GFP-PKBα, the basal as well as PDGF-stimulated Ser 473 phosphorylation decreased significantly, suggesting that this newly identified residue was critical for the stability of the Ser 473 phosphorylation (Figure 4B). This effect was not due to an impaired translocation of GFP-PKBα Q218A compared to GFP-PKBα wt upon PDGF stimulation (Figure 4B, bottom panel). Indeed, the level of Thr 308 phosphorylation upon stimulation was not modified significantly (Figure 4B, top panel). The recovery of Ser 473 phosphorylation upon pretreatment with okadaic acid prior to PDGF stimulation indicated that the loss of Ser 473 phosphorylation in GFP-PKBα Q218A mutant was due to an enhanced dephosphorylation (Figure 4C). A plausible explanation for this result was that the HM might be incorrectly positioned and thus was becoming more accessible to phosphatases.

In order to determine whether the Gln 218 Ala mutation affected the binding of AKT inhibitor VIII, the effect of an optimal (5 μM) and suboptimal (1 μM) dose of inhibitor VIII on Thr 308 phosphorylation was tested (Figure 4D). The enhanced inhibition of Thr 308 phosphorylation in the Gln 218 Ala mutant at 1 μM inhibitor suggested that the HM and the inhibitor might compete for the same or a proximate binding site.

In summary, the position of the HM in the inactive and activated state of PKB is likely to be very similar. The interaction between the Phe 469 and the Trp 80 suggested that a role of the HM motif in the regulation of PKB prior to its translocation and Ser 473 phosphorylation could be envisioned. Furthermore, the C-terminal HM and AKT inhibitor VIII could overlap at a common binding site.

**Figure 5:**
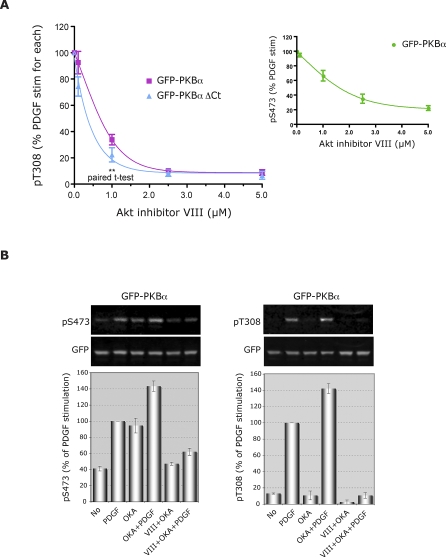
The Loss of Ser 473 Phosphorylation in PKBα upon AKT Inhibitor VIII Binding Is Due to Steric Hindrance (A) NIH3T3 cells expressing GFP-PKBa;pha; wt or GFP-PKBα ΔCt, deleted from the C-terminal HM motif (last 39 amino acids), were treated for 30 min with different concentrations of AKT inhibitor VIII prior to 5-min PDGF stimulation (stim) as indicated. Thr 308 and Ser 473 (only for wild type) phosphorylations were detected by western blot using phosphospecific antibodies, and the quantifications were performed using the Odyssey/LI-COR fluorescence system. For statistical analysis, a two-tailed paired *t*-test was done from six independent experiments. Double asterisks (**) indicate *p-*values < 0.001 at a concentration of 1 μM AKT inhibitor VIII. (B) NIH3T3 cells expressing wild-type GFP-PKBα were treated for 30 min with 5 μM of AKT inhibitor VIII (VIII) followed by 1 μM of okadaic acid (OKA) for a further 40 min prior to 5-min PDGF stimulation as indicated. Ser 473 (left panels) and Thr 308 (right panels) phosphorylations were detected by western blot using phosphospecific antibodies, and the quantifications were done using the Odyssey/LI-COR fluorescence system. The data are presented as a percentage of PDGF stimulation. Error bars indicate the standard error of the mean.

To determine whether the HM was implicated in AKT inhibitor binding as well as competing for the same (or a close) binding site, a deletion mutant of the last 39 residues of PKBα C-terminal part was made (Figure 5A). A dose-dependent inhibition of Thr 308 phosphorylation by AKT inhibitor VIII was performed on wild-type PKBα and mutant PKBα ΔCt. The deletion of the PKBα C-terminal part did not prevent the inhibition of Thr 308 phosphorylation by AKT inhibitor VIII. Moreover, a low dose of inhibitor (1 μM) slightly, but significantly, lowered Thr 308 phosphorylation in PKBα ΔCt compared to wild-type PKBα. This result showed that the binding of the inhibitor was not dependent on the C-terminal part of PKB and indicated further that the C-terminal HM and AKT inhibitor VIII could potentially be competing for a common or close binding site on the kinase domain.

To understand the mechanism involved in the loss of Ser 473 phosphorylation upon AKT inhibitor binding, experiments with okadaic acid and the inhibitor were performed (Figure 5B). Since the data in Figures 4D and 5A suggested that the HM and AKT inhibitor VIII could compete for the same binding site, the inhibitor binding could lead to an incorrect positioning of the HM, leading to an increased S473 dephosphorylation. A similar mechanism was shown above when mutating the residue Gln 218. Alternatively, the inhibitor binding could prevent the phosphorylation of Ser 473 by steric hindrance. As previously described [14], the inhibition of PP2A family of phosphatases by okadaic acid induced the phosphorylation of Ser 473 (but not Thr 308) to a similar extent as PDGF stimulation. However, the pretreatment with inhibitor VIII prior to okadaic acid, with and without PDGF, prevented the phosphorylation of Ser 473. This result suggested that loss of Ser 473 phosphorylation was likely to be due to the loss of accessibility of Ser 473 residue to upstream kinase(s) rather than a dephosphorylation.

### Conclusion

**Figure 6:**
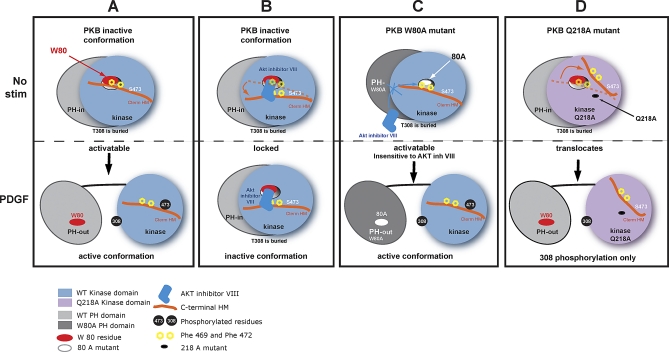
Mechanism of Inhibition of PKB Activity by AKT Inhibitor VIII (A) The upper panel shows PKBα in its inactive conformation (PH-in) prior to stimulation. The interaction of the PH domain (light grey) and the kinase domain (light blue) creates a PH domain-induced cavity in the kinase domain. The PH domain residue Trp 80 (red oval) interacts with the Phe 469 of C-terminal HM in orange through the PH-induced cavity. Both regulatory residues (Thr 308 and Ser 473) are unphosphorylated. Upon PDGF stimulation (lower panel), PKBα adopts its active (PH-out) conformation. PH and kinase domains separate, allowing the phosphorylation of the regulatory residues Thr 308 of the T-loop of the kinase domain and Ser 473 (black discs) on the HM. The PH domain-induced cavity closes up, and the HM residues Phe 469, Phe 473, and phosphorylated Ser 473 interact with the hydrophobic pocket. (B) The upper panel shows the binding of AKT inhibitor VIII (dark blue) to the Trp 80 through the PH-induced cavity of PKBα in its inactive conformation. The inhibitor and the C-terminal HM compete for binding of the same region. The dotted orange line shows the position of the C-terminal HM without inhibitor. The position of the C-terminal HM in presence of the inhibitor is represented as a solid orange line. The orange arrow represents the movement of the C-terminal. The lower panel depicts the locking of PKBα in the PH-in conformation. The binding of AKT inhibitor VIII prevents the change in conformation upon PDGF stimulation and Thr 308 from being phosphorylated. The phosphorylation of Ser 473 is prevented by a steric hindrance due to the binding of AKT inhibitor VIII in the same region as HM. (C) The mutation of the PH domain residue 80 to alanine (white oval) creates a PKBα insensitive to the inhibitor. Despite the preincubation with AKT inhibitor VIII, the drug fails to inhibit PDGF-induced change in conformation and activation of PKBα. (D) PKBα kinase domain residue Gln 218, positioned at close proximity to the C-terminal HM, was mutated to Ala (black spot). The mutation of this residue destabilises the positioning of the C-terminal HM, allowing Ser 473 to become more accessible to phosphatases. Upon PDGF stimulation, PKBα Q218A translocation to the plasma membrane and change in conformation allow the phosphorylation of Thr 308. However, Ser 473 remains dephosphorylated.

The novel findings from this study are summarised in Figure 6 (see figure legend). The molecular mechanism of interaction of the allosteric inhibitor AKT inhibitor VIII to a critical PH domain residue, Trp 80, was consistent with the model of binding through a PH-induced cavity formed in the kinase domain of PKB. This cavity was present in PKBα, whereas it was negligible in PKBγ. Chimera constructs of the PH and kinase domains suggested that the dimensions of the cavity were strongly correlated with the sensitivity to the inhibitor.

The in vivo FRET data showed that in the presence of AKT inhibitor VIII, PKB was maintained in its PH-in conformation. In this conformation, Thr 308 and Ser 473 phosphorylation were impaired. However, a difference in the inhibition of phosphorylation between the two sites was observed. The inhibition of phosphorylation on Thr 308 was linked to PKB being maintained in its PH-in conformation and not being accessible to PDK1.

Recently published in vitro data indicated that the binding of PKB PH domain to phosphoinositides did not seem to be affected by the inhibitor interaction [21]. However, the translocation of the GFP-PKB-mRFP reporter was prevented in presence of inhibitor. These results indicated that the maintenance of PKB at the plasma membrane appeared to require a change in conformation of the protein. The lack of PKB translocation could explain the loss of Ser 473 phosphorylation upon inhibitor binding. However, our results indicated that the inhibitor binding created a steric hindrance preventing the upstream kinase(s) from phosphorylating Ser 473.

We have addressed the possible molecular mechanisms involved in the regulation of the PKB inactive conformation by examining intramolecular domain interaction dynamics. The discovery of a PH-kinase domain interface in relation to the C-terminal HM was essential in the understanding of the role of PKB quaternary structure in the control of this critical regulator. Yet, the role played by the flexible linker that joins the PH and the kinase domains close to this interface, might also be important to comprehend fully this regulation.

In addition, we have depicted the possible molecular mechanism involved in the association of a new class of selective allosteric inhibitors that to date had remained elusive. We anticipate this will facilitate the optimisation of a new generation of more potent or more selective inhibitors.

## Materials and Methods

### Materials and antibodies.

Okadaic acid sodium salt and AKT inhibitor VIII were from Calbiochem (Merck KGaA). Human PDGF was from R&D Systems. Phospho-Akt Thr-308 (both rabbit polyclonal and monoclonal) and Ser-473, as well as pan Akt, were from Cell Signaling (New England BioLabs UK). Anti-GFP antibodies were in-house monoclonals. Other chemicals were from Sigma-Aldrich. Glass-bottom microwell culture dishes (35 mm; MatTek dishes) were from MatTek Corporation. The Odyssey blocking buffer was from LI-COR UK. The IRdye 800 anti-rabbit and IRdye 700 anti-mouse were used as secondary antibodies for the LI-COR/Odyssey system (from Rockland Incorporated).

### Fusion constructs design and direct site mutagenesis.

pEGFP-HA-Akt construct was described previously (murine AKT1; Genbank accession number NM_009652) [23]; to simplify, we called this construct GFP-PKBα.

GFP-PKB-mRFP was created by putting PCR-amplified mRFP (EcoRI-XbaI) instead of yellow fluorescent protein (YFP) in the vector pCDNA3-GFP-PKB-YFP (described previously [14,24]), using the oligonucleotides mRFP-Ct sense: AGAGAATTCGCCTCCTCCGAGGA CGTCATCAAGGAGTTCATGCGCTTCAAGG and mRFP-Ct antisense: ATCTCTAGA TTATGCACCGGTGGAGTGGCGGCCCTCGGCGCGCTCG TACTGTTCC.

Note that an extra XbaI site had to be removed using the Quick Change mutagenesis kit from Stratagene in the pCDNA3-GFP-PKB-YFP construct before the subcloning of mRFP (EcoRI-XbaI). The W80A mutation on GFP-PKB-mRFP was performed by Maria Deak from Dario Alessi's laboratory (Dundee, UK).

GFP-PKBα ΔCt was created by PCR amplification of GFP-PKBα without the C-terminal part, using the oligonucleotides AKT1 ΔCt-sense**:** GGCATGGACGAGCT GTACAAGGGT and AKT1 ΔCt antisense: TACGGATCCTCACTCCTCATCGAAATACCTGG.

The PCR amplification was then placed instead of wild-type PKBα in pEGFP-HA-Akt (GFP-PKBα) construct using SalI-BamHI restriction sites.

GFP-PKBγ was created by PCR amplification of PKBγ from pCDNA3-Myr HA AKT3 human (Addgene plasmid 9017). The oligonucleotides used for the amplification were PCR-PKBγ sense: GTCGACATGTACCCATACGATGTTCCAGATTACGCTTCCAGAATGAGCGATGTTACCATTGTGAAAGAAGGTTGG and PCR-PKBγ antisense: GGATCCTTATTCTCGTCCACTTGCAGA GTAGGAAAATTGAGGG. Prior to the PCR amplification of PKBγ, an internal BamHI site was removed in pCDNA3-Myr HA AKT3 by direct site mutagenesis using: PKBγ-BamHI-less sense: CTTTCAGGGCTCTTGATAAAGGACCCAAATAAACGCCTTGG and PKBγ-BamHI-less antisense: CCAAGGCGTTTATTTGGGTCC TTTATCAAGAGCCCTGAAAG. The PCR product was subcloned into the intermediate cloning vector zero blunt TOPO from Invitrogen, then excised using SalI-BamHI restriction enzymes. The excised PCR amplification was then put in phase in C-terminus of GFP (instead of PKBα in the vector GFP-PKBα cut with SalI-BamHI.

The chimera of the PH domain and linker of PKBα in fusion with the kinase domain and C-terminal of PKBγ (GFP-CHIM PHα-KINγ) was done first by creating a modified GFP-PKBα construct containing a BamHI site using the oligonucleotides AKT1-BamON-sense: GGGGCTGAAGAGATGGAGGGATCCCTGGCCAAGCCCAAGCAC and AKT1-BamON-antisense: GTGCTTGGGCTTGGCCAGGGAT CCCTCCATCTCTTCAGCCCC.

A PCR amplification of PKBγ kinase domain was then performed using the oligonucleotides ChimAKT3-sense: AGTGGATCCCTGGCCAAGCCCAAGCACAGAAAGA CAATGAATGATTTTGACTATTTG and ChimAKT3-antisense: ACTGGATCCTTA TTCTCGTCCACTTGCAGAGTAGG

The PCR product was cut with BamHI and inserted in the modified GFP-PKBα vector containing a BamHI site.

The mutants of PKB were obtained by direct site mutagenesis using the Quick Change mutagenesis kit from Stratagene using the oligonucleotides PKBα-W80A sense: CATCCGCTGCCTGCAGGCGACCACAGTCATTGAGCGC; PKBα-W80A antisense: GCGCTCAATGACTGTGGTCGCCTGCAGGCAGCGGATG; PKBα-Q218A sense: CGGCCCTCAAGTACTCATTCGCGACCCACGACCGCCTCTGC; PKBα-Q218A antisense: GCAGAGGCGGTCGTGGGTCGCGAATGAGTACTTGAGGGCCG; PKBγ-W79A sense: CAGATGTCTCCAGGCGACTACTGTTATAGAGAGAACATTTCATG; and PKBγ-W79A-antisense: CATGAAATGTTCTCTCTATAACAGTAGTCGCCTGGAGACATCTG.

### Cell culture and transfections.

NIH3T3 cells from ATCC were maintained in DMEM 10% Donor Calf Serum (DCS) and seeded at 150,000 in a well of a six-well plate or in a MatTek dish. The transfection was done with 2 μg of DNA of the different constructs (2+2 μg for cotransfections) using Lipofect AMINE/PLUS reagent (GIBCO-BRL) in OPTIMEM medium (GIBCO-BRL) as recommended by the manufacturer. The cells were left for 3 h in the transfection mix, and then the medium was removed and replaced with DMEM 10% DCS. The experiments were performed 24 or 48 h after transfection.

### Lysis conditions.

After stimulation or treatment as indicated, the cells were lysed for 15 min on ice in lysis buffer (20 mM Tris-HCl [pH 7.4], 150 mM NaCl, 100 mM NaF, 10 mM Na_4_P_2_O_7_, 10 mM EDTA supplemented with COMPLETE protease inhibitor cocktail tablet [Roche]). To terminate the reaction, 4× SDS sample buffer (125 mM Tris-HCl [pH 6.8], 6% SDS, 20% glycerol, 0.02% bromophenol blue supplemented with 10% β-mercaptoethanol) was added, and the samples boiled for 5 min. The proteins were separated on a 10% SDS-PAGE gel.

### Western blot analysis (classical ECL).

After electrophoresis, the polyacrilamide gels were transferred onto PVDF membrane (Immobilon P; Millipore) and incubated in blocking buffer TBS-T (10 mM Tris-HCl [pH 7.4], 150 mM NaCl, 0.05% Tween-20) supplemented with 3% BSA for 1 h. The membranes were then incubated with the different antibodies: phospho-Akt (Thr-308) antibody (rabbit polyclonal from Cell Signaling), phospho-Akt (Ser-473) (rabbit polyclonal antibody raised against the C-terminus phospho-peptide HFPQF*p*SYSASS of Akt1) or with the pan AKT at 1:1,000 for 1 to 3 h in TBS-T/3% BSA. The membranes were then washed in TBS-T with 0.2% Tween-20 and 1% milk for 1 h. The secondary HRP-antibodies were used at 1:5,000 in TBS-T with 5% milk for 1 h. Western blots were revealed by incubation with ECL (Amersham Biosciences). Density analysis of bands was done with NIH ImageJ 1.33u (U.S. National Institutes of Health, http://rsb.info.nih.gov/ij/). The analysis was performed by subtracting the background of the autoradiography and normalised as indicated on the graphs.

### Western blot (LI-COR/Odyssey fluorescence system).

After electrophoresis, the polyacrilamide gels were transferred onto PVDF membrane (Immobilon FL; Millipore), incubated in the Odyssey blocking buffer for 1 h, and then with the different antibodies phospho-Akt (Thr-308) antibody (rabbit polyclonal or monoclonal from Cell Signaling) or phospho-Akt (Ser-473) (rabbit polyclonal antibody raised against the C-terminus phospho-peptide HFPQF*p*SYSASS of Akt1) at 1:1,000 together with an anti-GFP in-house monoclonal at 1:2,500 for 4 h in Odyssey blocking buffer. The membranes were then washed in PBS 0.2% Tween-20 with 1% odyssey buffer for 1 h. The secondary antibodies IRdye 800 anti-rabbit and IRdye 700 anti-mouse (Rockland) were used at 1:2,500 and 1:5,000, respectively, in Odyssey blocking for 1 h. The blots were scanned with the infrared LI-COR scanner, allowing for simultaneous detection of two targets (anti-phospho and total protein) in the same experiment. For each experiment, the values of the phospho bands were normalized for the total amount of protein in each lane.

### Förster resonance energy transfer (FRET) by two-photon fluorescence lifetime imaging microscopy (FLIM).

NIH3T3 cells were seeded at 150,000 on 35-mm glass-bottom tissue culture dishes (MatTek) and transfected as described above. Twenty-four hours after transfection, the cells were treated in DMEM containing 10% DCS. The cells were washed twice with PBS, and then fixed in 4% paraformaldehyde in PBS for 10 min. The dishes were washed twice with PBS, and then 2 ml of PBS supplemented with 2.5% (w/v) DABCO as an antifade was added to the dishes. The images were acquired straightaway or stored at 4 °C.

Details about the method to detect FRET by time domain FLIM was as previously described [14,25]. We used a nonparametric Mann-Whitney test to compare the medians of the two datasets for the two-photon FLIM data using GraphPad InStat software (version 3.0 for Mac-2001). To interpret the distribution of data, box and whisker plots were used. The box and whisker plot is a histogram-like method for displaying upper and lower quartiles, and maximum and minimum values in addition to median.

### Molecular modelling.

In order to check the validity of the models, we performed several molecular dynamics simulations. Vacuum calculations were performed on an SGI Octane workstation using InsightII and Discover ver. 2000 (Accelrys). The hydrated models calculations were performed using NAMD [26] software v2.6 and CHARMM 27 force-field. All simulations were performed on a 32-processor IBM PC-cluster using 8 to 16 processors. The simulation boxes were built using VMD v1.8.6 facilities. All images were captured from VMD [27] using the internal ray-tracing software Tachyon.

Two models were built starting from the model of the PH/KIN PKBα complex previously described [14]. The whole complex was immersed in a box of water, fully ionized, with all charges neutralized by counter ions, and salt was added to reach the biological concentration of 150 mM. The wild type was built first, and the W80 was subsequently mutated into A80.

Model 1 (wild type) totalled 47,114 atoms with 13,394 water molecules (TIP3P) in a box of 88.94 × 73.99 × 77.87 Å^3^, at *T* = 300 K and *P* = 1 bar. Time of simulation: 6.22 ns.

Model 2 (W80A) totalled 47,103 atoms with 13,395 water molecules (TIP3P) in a box of 88.94 × 73.99 × 77.87 Å^3^, at *T* = 300 K and *P* = 1 bar. Time of simulation: 6.13 ns.

During the two simulations, a water cavity opened quickly (after about 0.2 ns) just in front of residue 80 going from the outer surface of the protein directly to the surface of residue 80.

For wild-type (PKBα W80), the cavity was stable and almost as wide as deep. W80 was located about 8–8.5 Å from the outer surface of the protein, and the cavity had a maximum opening of around 8–9 Å. The cavity was full of water (see Figure 2).

For the W80A mutant, the cavity appeared more unstable with fluctuations and was deeper than it was wide. A80 was located 14–15 Å from the protein surface (compared to 8–8.5 Å in the wild type), and the maximum opening was around 6–7 Å. The cavity was again full of water.

### Positioning of the C-terminal.

The final model of the wild-type PH/KIN PKBα complex after molecular dynamics simulation was superimposed (kinase domain only) over the published X-ray structure of AKT-2 (PKBβ) [22] (PDB codes: 1O6K and 1O6L). After superimposition, the kinase domain of PKBβ was deleted, leaving the C-terminal (466–479) of PKBβ lying on the surface of the PH/KIN complex of PKBα. The C-terminal was then mutated to correspond to the sequence of the C-terminal of PKBα, as below:

1O6K:QRTHFPQF**D**YSASI

PKBα:RRPHFPQFSYSASG

Underlined residues were mutated during the building (only three residues). The bold and underlined S474D mutation, which was introduced in PKBβ [16], was also present (as S473D) in the first model of PKBα. However, in a second step, the reverse mutation D473S was built in order to get back to the wild-type model.

With the D473 mutation, a first model was built in a box of dimensions: 78.74 × 91.28 × 73.66 Å^3^, at *T* = 300 K and *P* = 1 bar, with a total amount of 48,537 atoms of which 13,793 were water molecules, simulation time: 3.43 ns. During the simulation, an interaction between R465 and D473 was observed due to the fact that the C-terminal was truncated on its N-terminus. Thus the N-terminal of the C-terminal was moved toward the surface of the protein away from D473, and a second model was built: 79.52 × 91.79 × 73.82 Å^3^, at *T* = 300 K and *P* = 1 bar, with a total amount of 49,619 atoms of which 14,153 water molecules, simulation time: 7.2 ns.

With the reverse D473S mutation, one model was built: 78.74 × 91.28 × 73.66 Å^3^, at *T* = 300 K and *P* = 1 bar, with a total amount of 48,531 atoms of which 13,872 were water molecules, simulation time: 11.2 ns.

Even though the C-terminal is an isolated short peptide strand, it displays a sustainable stability for the duration of the various molecular dynamics runs. This is due to many close contacts and hydrogen bonds with mainly the kinase domain. There are hydrogen bondings of the C-terminal backbone with several polar groups of the kinase domain: F472 with S216, F469 with Q218, P467 with T219, and a strong salt bridge between R465 and E184. A full p-stacking (face to face) between the F462 and F225 residue is observed. Very close contacts between the C-terminal and the following residues in the kinase domain: K183, V187, A188, H220, D221, and L223 are also observed. It is of note that when the C-terminal was added to the PH/KIN complex, the number of salt bridges between the PH and kinase domains increased from seven bridges for the PH/KIN complex to ten bridges for the full PH/KIN/C-term complex. This implied a stabilization of the AKT complex upon binding of the C-terminal.

### AKT inhibitor VIII binding simulation.

The starting point was the previously equilibrated model of the PH/KIN complex of human PKBα with the cavity filled with water molecules. The inhibitor was carefully docked within the water cavity. The complex was submitted to a simulated annealing procedure to get the best docking solution using the Affinity module within Insight software. Initially, random docking was performed with a flexible ligand without electrostatic interactions and with Van der Waals interactions reduced to 10%. At a second stage, the best solutions were submitted to a simulated annealing (SA) procedure with all nonbonded interactions on all free protein side-chains and the tethered backbone. Fifty stages of SA of 100 fs each were performed from 500 to 300 K before full minimization (1,000 steps). The best solution was submitted to several 1-ns molecular dynamics without any constraints. In all cases, the benzimidazolone moiety located at the extremity of the inhibitor was associated by hydrogen bonding to the residues Q218, T219, or S216. The imidazolo-quinoxaline moiety remained in close contact with the key residue W80 (Figure S2). The aromatic part of the inhibitor, which was embedded inside the cavity, was surrounded by a hydrophobic core containing: I186, F225, and H194 residues, as well as the W80.

## Supporting Information

Figure S1 Molecular Dynamic Simulation of AKT Inhibitor VIII in PKBα PH-in The dynamic structure of the PKBα PH domain (pink) and the reconstructed kinase domain (green) is shown in complex (PKBα PH-in conformer). The secondary structure of the kinase domain is represented as a white ribbon. The PH domain residue Trp 80 is shown in red. The position of AKT inhibitor VIII (blue) is presented following a dynamic run of 1 ns. The inhibitor is deeply and stably embedded in the cavity of wild-type PKBα PH-in conformation. A distance of 4 Å (between the two N) or less than 3 Å (between H and N) can be measured from the quinoxalin head of AKT inhibitor VIII to the Trp 80, suggesting a possible interaction by hydrogen bonding. The kinase domain residue Q218 highlighted in yellow is positioned at less than 5 Å from AKT inhibitor VIII.
(2.15 MB PDF)

Figure S2 Distance between the Nitrogen Atom of the Imidazolo-Quinoxaline Moiety of Inhibitor VIII and the Nitrogen of the Indole Nucleus of Trp 80 upon Time (411 KB PDF)

## Abbreviations

FLIM: fluorescence lifetime imaging microscopy
FRET: Förster resonance energy transfer
GFP: green fluorescent protein
HM: hydrophobic motif
mRFP: monomeric red fluorescent protein
PDGF: platelet-derived growth factor
PDK1: 3-phosphoinositide-dependent protein kinase-1
PH: pleckstrin homology
PH-in: protein kinase B in an inactive conformation
PKB: protein kinase B

## References

[pbio-1000017-b001] Hill MM, Hemmings BA (2002). Inhibition of protein kinase B/Akt. Implications for cancer therapy. Pharmacol Ther.

[pbio-1000017-b002] Nicholson KM, Anderson NG (2002). The protein kinase B/Akt signalling pathway in human malignancy. Cell Signal.

[pbio-1000017-b003] Kim D, Dan HC, Park S, Yang L, Liu Q (2005). AKT/ PKB signaling mechanisms in cancer and chemoresistance. Front Biosci.

[pbio-1000017-b004] Brazil DP, Hemmings BA (2001). Ten years of protein kinase B signalling: a hard Akt to follow. Trends Biochem Sci.

[pbio-1000017-b005] Cameron AJ, De Rycker M, Calleja V, Alcor D, Kjaer S (2007). Protein kinases, from B to C. Biochem Soc Trans.

[pbio-1000017-b006] Alesi DR, James SR, Downes CP, Holmes AB, Gaffney PR (1997). Characterization of a 3-phosphoinositide-dependent protein kinase which phosphorylates and activates protein kinase Balpha. Curr Biol.

[pbio-1000017-b007] Sarbassov DD, Guertin DA, Ali SM, Sabatini DM (2005). Phosphorylation and regulation of Akt/ PKB by the rictor-mTOR complex. Science.

[pbio-1000017-b008] Feng J, Park J, Cron P, Hess D, Hemmings BA (2004). Identification of a PKB/Akt hydrophobic motif Ser-473 kinase as DNA-dependent protein kinase. J Biol Chem.

[pbio-1000017-b009] Alessi DR, Andjelkovic M, Caudwell B, Cron P, Morrice N (1996). Mechanism of activation of protein kinase B by insulin and IGF-1. EMBO J.

[pbio-1000017-b010] Lasserre R, Guo XJ, Conchonaud F, Hamon Y, Hawchar O (2008). Raft nanodomains contribute to Akt/ PKB plasma membrane recruitment and activation. Nat Chem Biol.

[pbio-1000017-b011] Milburn CC, Deak M, Kelly SM, Price NC, Alessi DR (2003). Binding of phosphatidylinositol 3,4,5-trisphosphate to the pleckstrin homology domain of protein kinase B induces a conformational change. Biochem J.

[pbio-1000017-b012] Thomas CC, Deak M, Alessi DR, van Aalten DM (2002). High-resolution structure of the pleckstrin homology domain of protein kinase b/akt bound to phosphatidylinositol (3,4,5)-trisphosphate. Curr Biol.

[pbio-1000017-b013] Komander D, Fairservice A, Deak M, Kular GS, Prescott AR (2004). Structural insights into the regulation of PDK1 by phosphoinositides and inositol phosphates. EMBO J.

[pbio-1000017-b014] Calleja V, Alcor D, Laguerre M, Park J, Vojnovic B (2007). Intramolecular and intermolecular interactions of protein kinase B define its activation in vivo. PLoS Biol.

[pbio-1000017-b015] Gao T, Furnari F, Newton AC (2005). PHLPP: a phosphatase that directly dephosphorylates Akt, promotes apoptosis, and suppresses tumor growth. Mol Cell.

[pbio-1000017-b016] Yang J, Cron P, Thompson V, Good VM, Hess D (2002). Molecular mechanism for the regulation of protein kinase B/Akt by hydrophobic motif phosphorylation. Mol Cell.

[pbio-1000017-b017] Barnett SF, Bilodeau MT, Lindsley CW (2005). The Akt/ PKB family of protein kinases: a review of small molecule inhibitors and progress towards target validation. Curr Top Med Chem.

[pbio-1000017-b018] Lindsley CW, Zhao Z, Leister WH, Robinson RG, Barnett SF (2005). Allosteric Akt ( PKB) inhibitors: discovery and SAR of isozyme selective inhibitors. Bioorg Med Chem Lett.

[pbio-1000017-b019] Zhao Z, Leister WH, Robinson RG, Barnett SF, Defeo-Jones D (2005). Discovery of 2,3,5-trisubstituted pyridine derivatives as potent Akt1 and Akt2 dual inhibitors. Bioorg Med Chem Lett.

[pbio-1000017-b020] Logie L, Ruiz-Alcaraz AJ, Keane M, Woods YL, Bain J (2007). Characterization of a protein kinase B inhibitor in vitro and in insulin-treated liver cells. Diabetes.

[pbio-1000017-b021] Green CJ, Goransson O, Kular GS, Leslie NR, Gray A (2008). Use of Akt inhibitor and a drug-resistant mutant validates a critical role for PKB/Akt in the insulin-dependent regulation of glucose and system A amino acid uptake. J Biol Chem.

[pbio-1000017-b022] Yang J, Cron P, Good VM, Thompson V, Hemmings BA (2002). Crystal structure of an activated Akt/protein kinase B ternary complex with GSK3-peptide and AMP-PNP. Nat Struct Biol.

[pbio-1000017-b023] Watton SJ, Downward J (1999). Akt/ PKB localisation and 3' phosphoinositide generation at sites of epithelial cell-matrix and cell-cell interaction. Curr Biol.

[pbio-1000017-b024] Calleja V, Ameer-Beg SM, Vojnovic B, Woscholski R, Downward J (2003). Monitoring conformational changes of proteins in cells by fluorescence lifetime imaging microscopy. Biochem J.

[pbio-1000017-b025] Alcor D, Calleja V, Larijani B, Larijani B, Woscholski R, Rosser CA, Walker JM (2009). Revealing signalling in single cells by single and two-photon fluorescence lifetime imaging microscopy. Methods in molecular biology.

[pbio-1000017-b026] Phillips JC, Braun R, Wang W, Gumbart J, Tajkhorshid E (2005). Scalable molecular dynamics with NAMD. J Comput Chem.

[pbio-1000017-b027] Humphrey W, Dalke A, Schulten K (1996). VMD: visual molecular dynamics. J Molc Graph.

