# Supplementary material for: Role of a Novel PH-Kinase Domain Interface in PKB/Akt Regulation: Structural Mechanism for Allosteric Inhibition
Source: PLoS Biol. 2009 Jan 20;7(1):e1000017. doi: 10.1371/journal.pbio.1000017 (PMC2628406; doi:10.1371/journal.pbio.1000017)
Supplement: Supplementary file 1 [file pbio.1000017.sg001.pdf]

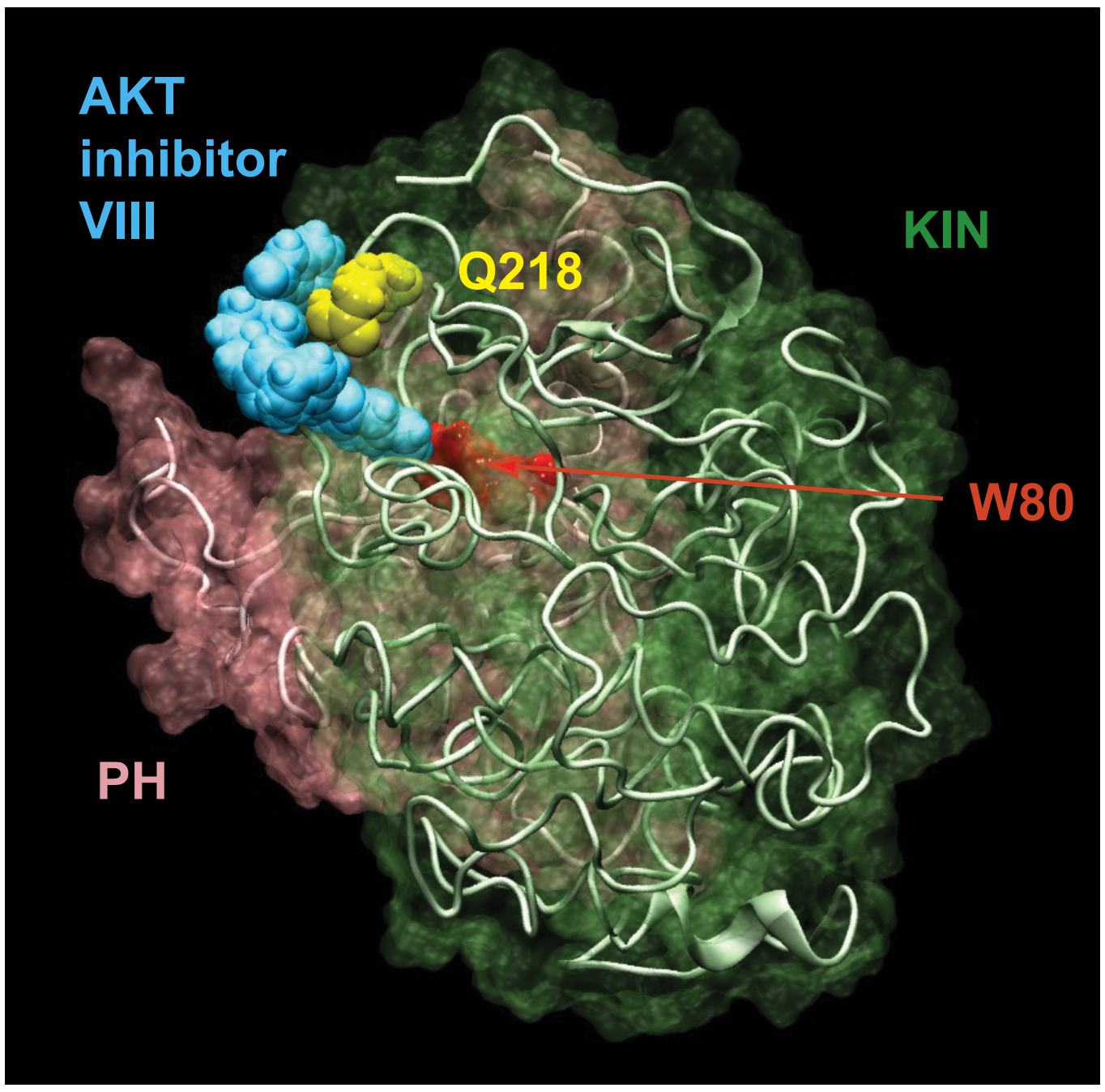
AKT
inhibitor
VIII
Q218
KIN
W80
PH
