# Supplementary material for: Role of a Novel PH-Kinase Domain Interface in PKB/Akt Regulation: Structural Mechanism for Allosteric Inhibition
Source: PLoS Biol. 2009 Jan 20;7(1):e1000017. doi: 10.1371/journal.pbio.1000017 (PMC2628406; doi:10.1371/journal.pbio.1000017)
Supplement: Supplementary file 2 [file pbio.1000017.sg002.pdf]

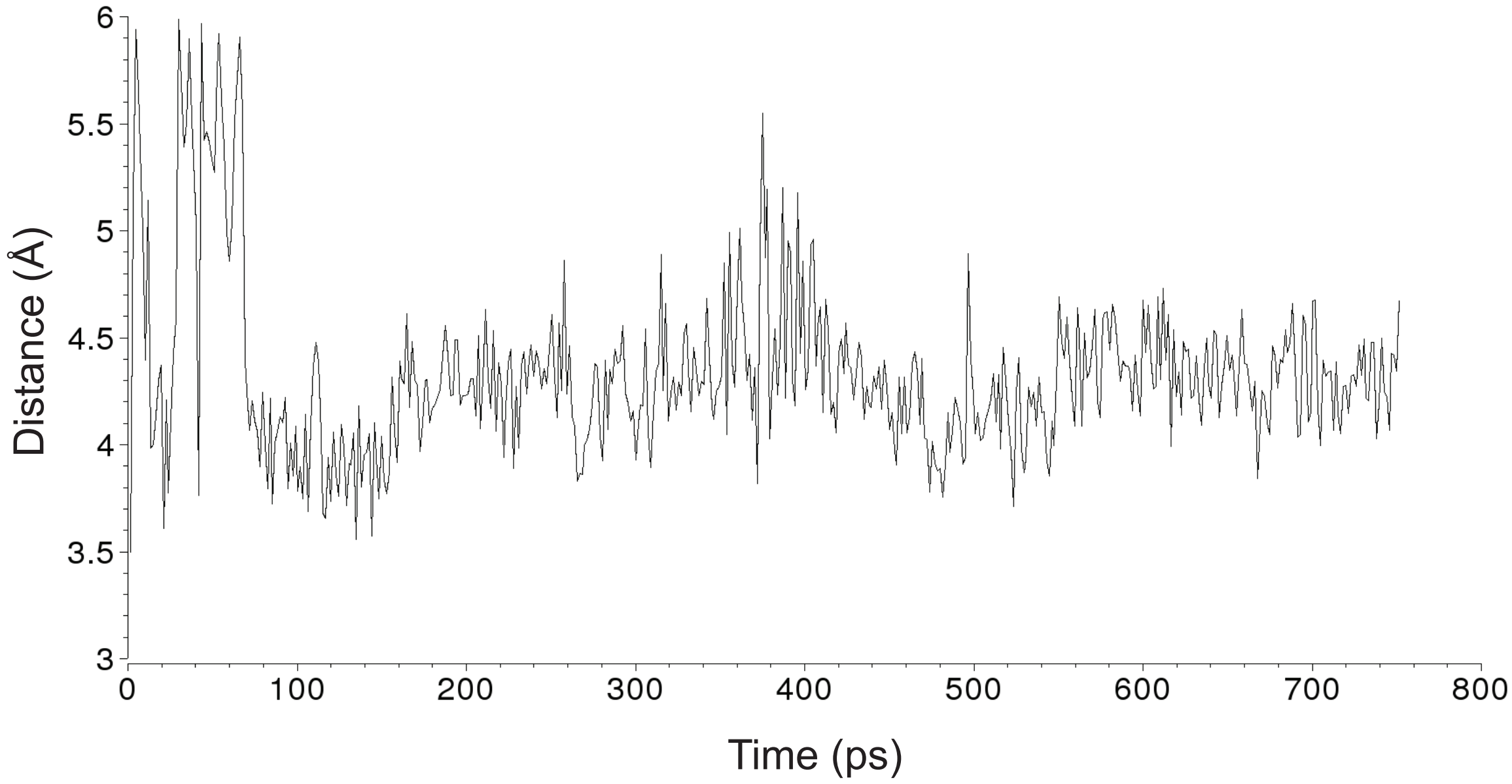

Distance (Å)
6
5.5
5
4.5
4
3.5
3
0
100
200
300
400
500
600
700
800
Time (ps)
